# Theological Students

**Published:** 1856-12

**Authors:** 


### THEOLOGICAL STUDENTS,

With warm hearts and eager expectations, feed on the hopes of good to be done by them, when they have left the seminary.

In their anxieties, not to be found working *with untempered mortar*, and to make of themselves laborers, *who need not be ashamed*, and that from unfaithfulness or incompetency *the blood of souls may not rest on the hem of their garments*—in these solicitudes, they very often apply themselves so sedulously to study, that they have not time for taking an amount of exercise, absolutely essential to the safety of their constitutions. We remember the names of many, who, in our schoolboy days, considered every hour out of their study, so much time lost, and they have died, long, long ago. As for ourselves, we let Venable, and Clelland, and Allen, and the lamented Congressman, J. G. Miller, study for us, while we "knocked around," and here we are yet, on *this* side of forty-five, our eyes not spectacled, our head not bald, nor hair gray. We remember, however, several things connected with our college course. We lived by system. Got up at daylight, were never a moment behind time at recitation, never out of bed at ten o'clock, except on Society nights, and were always in a good humor, *because* we were never out of money—that affliction came on later. Now, with system, regular habits, moderate study, good humor, and money, what collegian could get sick, even if he were to try? And there was our friend, K. H. A., whom we found reciting in the senior class, when we entered college with the junior, and on graduating two years later, under that great orator and Christian gentleman, Gideon Blackburn, left him in the preparatory department. He never studied at all, took the world easy, never was sick a minute, and although he never got through college, he got out of it, wriggled himself into the ministry, got a wife, a fortune, and *then* a D.D., and has made one of the really useful men of his time. Now, we consider that as infinitely better than studying oneself to death at the threshold of the pulpit.

We believe that the first duty of a theological student is to take care of his health, and chew Hebrew roots and Greek themes afterwards.

As to one of our seminaries, nearly one-fifth of two specified classes died before the completion of their professional studies. To the friends of religion, this is an alarming statement, and in view of it, we call attention to the subject, with the suggestion, that lectures on the general laws of health, by educated physicians of long practice, should form a part of the course of study during each session, from the first Freshman year at college, until they leave the seminary; and then, instead of a race of sickly imbeciles, as to bodily health, our pulpits would be filled with giants in intellect and physical vigor.

